# Effects of tadalafil treatment after bilateral nerve-sparing radical prostatectomy: quality of life, psychosocial outcomes, and treatment satisfaction results from a randomized, placebo-controlled phase IV study

**DOI:** 10.1186/s12894-015-0022-9

**Published:** 2015-04-12

**Authors:** Hitendra R Patel, Dapo Ilo, Nimish Shah, Béatrice Cuzin, David Chadwick, Robert Andrianne, Carsten Henneges, Jane Barry, Katja Hell-Momeni, Julia Branicka, Hartwig Büttner

**Affiliations:** Department of Urology, University Hospital North Norway, Sykehusvegen 38, 9038 Tromsø, Norway; Lilly UK, Basingstoke, UK; Addenbrooke’s Hospital, Cambridge, UK; Department of Urology, Edouard Herriot University Hospital, Lyon, France; South Tees Hospitals NHS Foundation Trust, Stockton-on-Tees, UK; Centre Hospitalier Universitaire de Liège, Service d’Urologie, Belgium; Lilly Deutschland GmbH, Bad Homburg, Germany; Eli Lilly Polska, Warsaw, Poland

**Keywords:** PDE5 inhibitor, Tadalafil, Erectile dysfunction, Randomized clinical trial, Prostate cancer, Prostatectomy, Quality of life, Urinary incontinence

## Abstract

**Background:**

This multicenter, randomized, double-blind, double-dummy, placebo-controlled trial primarily evaluated the efficacy of tadalafil once-daily (OaD) or on-demand (“pro-re-nata”; PRN) treatment, started early post-nsRP. Secondary outcome-measures on quality-of-life (QoL) and treatment satisfaction are reported.

**Methods:**

Patients, aged <68 yrs, with adenocarcinoma of the prostate (Gleason ≤ 7, normal preoperative erectile function [EF]) were randomized post-nsRP 1:1:1 to 9-month treatment with tadalafil 5 mg OaD, tadalafil 20 mg PRN, or placebo, followed by 6-week drug-free washout and 3-month open-label tadalafil OaD treatment (OLT). The main outcome measures were Changes in Expanded Prostate Cancer Index Composite (EPIC-26), Erectile Dysfunction Inventory of Treatment Satisfaction (EDITS), and Self-Esteem and Relationship (SEAR) questionnaires (mixed-model-for-repeated-measures, including terms for treatment, visit, treatment-by-visit interaction, age-group, country, baseline-score). LS means with 95% confidence interval (CI) are reported.

**Results:**

423 patients were randomized to 3 treatment-groups: tadalafil OaD (N = 139), PRN (N = 143), or placebo (N = 141). In each group, 57 (41.0%), 58 (40.6%), and 50 (35.5%) patients were aged 61-68 yrs. At the end of double-blind treatment (DBT), patients’ EPIC sexual domain-scores improved significantly with tadalafil OaD versus placebo (treatment effect [95% CI]: 9.6 [3.1,16.0]; p = 0.004); comparisons of PRN versus placebo at end of DBT, and comparisons of tadalafil OaD and PRN versus placebo after OLT were not significant. Only in older patients (61-68 yrs; age-by-treatment p ≤ 0.1), EPIC urinary incontinence domain-scores also improved significantly with tadalafil OaD versus placebo (overall treatment effect across all visits, 8.3 [0.4,16.1]; p = 0.040). Treatment satisfaction increased significantly in both tadalafil groups, EDITS total-scores increased significantly with OaD and PRN versus placebo during DBT (p = 0.005 and p = 0.041, respectively). At the end of OLT, improvement was significant for tadalafil OaD versus placebo only (p = 0.035). No significant differences were observed for SEAR.

**Conclusions:**

These results suggest that chronic dosing of tadalafil improves QoL of patients post-nsRP. The improvement of urinary incontinence in elderly patients randomized to tadalafil OaD may contribute to this effect.

**Trial registration:**

www.clinicaltrials.gov, NCT01026818.

**Electronic supplementary material:**

The online version of this article (doi:10.1186/s12894-015-0022-9) contains supplementary material, which is available to authorized users.

## Background

Erectile dysfunction (ED) is a common problem after nerve-sparing radical prostatectomy (nsRP) for localized prostate cancer [1,2]. First-line therapy for ED after nsRP is the use of a phosphodiesterase type 5 (PDE5) inhibitor [3]. Alternative treatment options include intracavernosal injections, e.g., of the “trimix” combination of alprostadil, phentolamine, and papaverine, intrautheral alprostadil suppositories, vacuum erection devices, or inflatable penile prosthesis [3]. PDE5 inhibitors are generally well-tolerated and effective treatments of ED after nsRP, although they are less effective in the post-nsRP population than in the general population [1,4,5]. There is still considerable debate as to which patients might benefit most from PDE5 inhibitor treatment after nsRP, and what would be the optimal time for treatment initiation, the optimal duration of treatment, and the most appropriate efficacy endpoints [6].

To date, there have been 4 randomized controlled trials evaluating the impact of the early use of PDE5 inhibitors in men with ED following nsRP. Nightly administration of sildenafil for 36 weeks, starting 4 weeks after surgery, markedly increased the return of normal spontaneous erections; the study was stopped early since it was expected not to meet its primary endpoint [7]. Vardenafil treatment for 9 months, starting within 2 weeks after surgery, was efficacious when used on-demand (pro-re-nata, PRN), but had no significant effect on unassisted erectile function (EF) after drug-free washout [5]. In a recent study, 3 months of treatment with avanafil 100 or 200 mg PRN significantly improved drug-assisted EF after prostatectomy, but a sustained effect on unassisted EF was not assessed [8]. Finally, the REACTT trial has evaluated the effect of the long-acting PDE5 inhibitor tadalafil, showing that tadalafil once daily [OaD] was most effective on drug-assisted EF during 9 months of double-blind treatment (DBT) [9]. The study suggested a potential role of tadalafil OaD provided early after surgery in contributing to EF recovery, and a significant protection from penile length loss, possibly by protecting from penile structural changes. However, also in this trial, unassisted EF was not improved after drug-free washout following DBT [9].

These post-nsRP studies have predominantly looked at standard ED outcomes, including the International Index of Erectile Function (IIEF) domain scores and Sexual Encounter Profile (SEP) questions. However, prostate cancer patients frequently report other associated symptoms, e.g., penile length loss, climacturia, or urinary incontinence, which may have a pronounced impact on their quality of life (QoL).

This manuscript addresses secondary outcome measures on QoL and treatment satisfaction in early post-nsRP patients who participated in the REACTT trial [9]. The QoL questionnaires used evaluate those aspects of QoL which are specifically relevant for prostate cancer patients and patients with ED, for example the Expanded Prostate Cancer Index Composite Short Form (EPIC-26) [10,11]. This questionnaire addressed 4 different domains — sexual, urinary (divided into incontinence and irritation/obstruction subscales), bowel, and hormonal function [10,11]. The sexual and urinary incontinence domain scores are the most relevant to QoL of post-nsRP prostate cancer patients, as ED and urinary incontinence are common sequelae of nsRP [12,13]; other subscales are more pertinent to prostate cancer patients who have had radiation or hormonal deprivation therapy. This study also used the Erectile Dysfunction Inventory of Treatment Satisfaction (EDITS) questionnaire to assess patient and partner satisfaction with ED treatment, and the Self-Esteem and Relationship (SEAR) instrument, which assessed patient and partner sexual relationship confidence and self-esteem [14,15].

## Methods

### Patients

All enrolled patients were adult men, aged < 68 years at the time of nsRP, with normal preoperative EF (IIEF-EF domain score ≥ 22) [9] who underwent nsRP for organ-confined, non-metastatic prostate cancer (Gleason score ≤ 7, prostate specific antigen <10 ng/mL). These patients were enrolled between November 2009 and August 2011, in 50 centers in 9 European countries and Canada; detailed trial design and eligibility criteria are available at www.clinicaltrials.gov (NCT01026818) and in [9]. The study was approved by the responsible ethical review boards (Additional file 1: Table S1).

### Trial design

This multicenter, Phase IV, randomized, double-blind, 3-arm, placebo-controlled, parallel-group trial was conducted in accordance with the declaration of Helsinki; appropriate ethical review boards approved the study protocol for each country. All patients signed written informed consent. The trial consisted of a screening period (including nsRP), 9 months of randomized, double-blind, double-dummy, treatment with tadalafil 5 mg OaD, tadalafil 20 mg PRN, or placebo, starting within 6 weeks after nsRP (double-blind treatment; DBT), 6 weeks of drug-free washout, and 3 months of open-label treatment with tadalafil 5 mg OaD (OLT, all patients) (Additional file 1: Figure S1). Matching placebo tablets identical to the 5 mg and 20 mg tadalafil tablets were used to ensure that the blinded regimen was identical for all treatment groups. During DBT, patients received tadalafil 5 mg OaD (+ placebo PRN), tadalafil 20 mg PRN (+ placebo OaD) or placebo (OaD + PRN). For PRN dosing, patients were permitted to take up to 3 tablets per week (and no more than 1 per day). During drug-free washout, patients received no study drug. During the open-label period, all patients received tadalafil 5 mg OaD.

### Main outcome measures

Patients’ EF was assessed using the IIEF-EF domain score at baseline (post-nsRP), the end of DBT, and the end of OLT [16].

EPIC-26 domain scores [10,11], with a special focus on the sexual and urinary incontinence domain scores, were used to assess patients’ prostate-specific QoL status at baseline, the end of DBT, and the end of OLT. In addition, partners were asked to complete the EPIC-26P questionnaire. Individual item and EPIC domain scores were standardized to a 0 to 100 scale; higher scores represent better QoL [10,11].

The 11-item EDITS questionnaire was used to assess patients’ treatment satisfaction at the end of DBT and OLT [14]. Responses were based on the 4 weeks preceding assessments. Each question was rated from 0 to 4 with higher scores indicating higher satisfaction, and the total score (average of the individual item scores) was reported.

The SEAR questionnaire was used to assess the patients’ sexual relationships and self-esteem at baseline, the end of DBT, and the end of OLT [15]. The SEAR questionnaire has 2 domains of sexual relationship (domain score range 8 to 40) and confidence (range 6 to 30), the latter of these domains can be divided into 2 subscales on self-esteem (range 4 to 20) and overall relationship (range 2 to 10) [15]. Higher scores indicate a more favorable response.

### Statistical analysis

The planned sample size of 412 patients was based on the primary outcome (proportion of patients achieving IIEF-EF ≥22) at the end of drug-free washout period [9]. All analyses were based on the intent-to-treat (ITT) population, which included all randomized patients with baseline data and at least 1 post-baseline visit. Pre-specified treatment group comparisons were tadalafil OaD versus placebo and tadalafil PRN versus placebo.

Changes from baseline in EPIC and SEAR domain scores, and actual EDITS total scores were assessed using a pre-specified mixed model for repeated measures (MMRM), assuming an unstructured covariance matrix and included terms for baseline domain score, treatment, country, visit, visit-by-treatment interaction, age group, and age-group-by treatment interaction (included only if p < 0.1). Least squares means (LS mean) changes from baseline and the associated 95% CIs were provided for the 2 key visits (end of DBT and end of OLT). In case of a significant age-group by treatment interaction (p < 0.1), the overall treatment effect (across all visits) by age group was also provided. Agreement between patient- and partner-rated EPIC scores was assessed using unweighted Cohen’s kappa statistics, which is adjusted for agreement by chance.

Spearman rank correlation coefficients and the associated 95% CIs were calculated post-hoc to assess correlations at baseline, end of DBT, and end of OLT between: (a) EPIC sexual and urinary incontinence domain scores; (b) IIEF-EF scores and EPIC sexual and urinary incontinence domain scores.

A 2-sided 5% level of significance was used for p-values for treatment group comparisons; a 10% level of significance was used for p-values for interaction terms. No other adjustments for multiplicity were applied for the analyses reported here. Data were analyzed using SAS 9.2 software (SAS Institute Inc., Cary, USA).

## Results

### Patient disposition and baseline characteristics

Of 583 patients screened, 423 were randomized to DBT, 422 were included in the ITT population: 139 (32.9%) patients were treated with tadalafil OaD, 142 (33.7%) with tadalafil PRN, and 141 (33.4%) with placebo (Additional file 1: Figure S2). Patient disposition, baseline demographics [12], and relevant disease characteristics were balanced in the 3 treatment groups (Table 1). As per inclusion criteria, all patients had IIEF-EF domain scores ≥22 before nsRP.

**Table 1:** **Baseline characteristics**

	**Tadalafil OaD (N = 139)**	**Tadalafil PRN (N = 143)** ^**a**^	**Placebo (N = 141)**	**Overall (N = 423)** ^**a**^
**Age, years**				
Mean (SD)	58.6 (5.07)	57.5 (5.91)	57.6 (5.69)	57.9 (5.58)
<61 years, n (%)	82 (59.0)	85(59.4)	91 (64.5)	258 (61.0)
61-68 years, n (%)	57 (41.0)	58 (40.6)	50 (35.5)	165 (39.0)
**nsRP approach, n (%)**				
Open surgery	68 (48.9)	65 (45.5)	56 (39.7)	189 (44.7)
Conventional laparoscopy	29 (20.9)	31 (21.7)	28 (19.9)	88 (20.9)
Robot-assisted laparoscopy	31 (22.3)	41 (28.7)	44 (31.2)	116 (27.4)
Other	11 (7.9)	6 (4.2)	13 (9.2)	30 (7.1)
**IIEF-EF at randomization (V4, after prostatectomy)**				
N with data	137	140	137	414
Mean (SD)	6.0 (5.80)	6.7 (5.57)	6.5 (6.08)	6.4 (5.81)
**EPIC sexual domain score**				
N with data	133	140	137	410
Mean (SD)	19.8 (19.56)	21.9 (20.16)	20.1 (21.87)	20.6 (20.53)
**EPIC urinary incontinence domain score**				
N with data	133	139	137	409
Mean (SD)	46.7 (30.71)	47.9 (28.89)	49.5 (28.05)	48.0 (29.17)
**EPIC urinary irritative/obstructive domain score**				
N with data	131	137	134	402
Mean (SD)	78.1 (18.92)	81.5 (15.08)	81.3 (16.42)	80.4 (16.88)
**EPIC bowel domain total score**				
N with data	129	134	136	399
Mean (SD)	88.3 (15.32)	91.2 (10.31)	89.9 (13.69)	89.8 (13.27)
**EPIC hormonal domain score**				
N with data	130	137	136	403
Mean (SD)	90.0 (12.71)	92.0 (10.51)	91.4 (10.89)	91.2 (11.39)

^a^Data presented for all patients randomized. One patient assigned to tadalafil PRN did not receive any study drug and was therefore not included in the ITT population.

*Abbreviations:* *EPIC* Expanded Prostate Cancer Index Composite (EPIC-26), *IIEF-EF* International Index of Erectile Function – Erectile Function, *ITT* intent-to-treat, *N* number of patients, *n* number of patients with characteristic, *nsRP* bilateral nerve-sparing prostatectomy, *NSS* Nelson Nerve-Sparing score, *OaD* once daily, *PRN* “pro-re-nata”/on-demand, *SD* standard deviation, *V* visit EPIC scores range from 0–100, higher scores indicate better values.

### EPIC domain scores – patient rating

**Figure 1:**
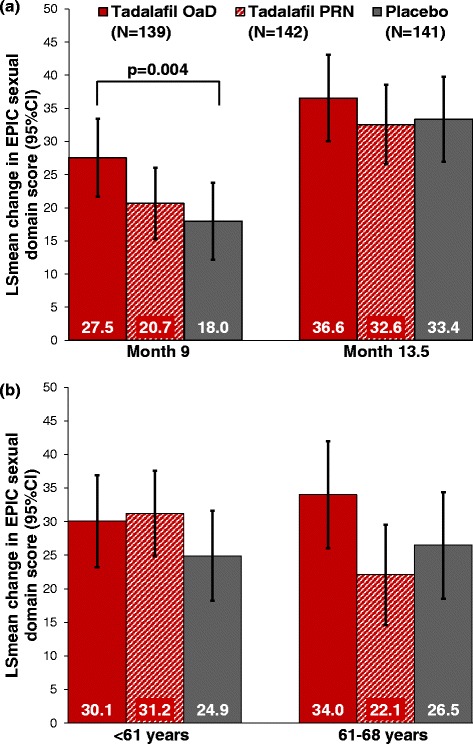
EPIC Sexual Domain Score Changes from Baseline: **(a)** at the end of DBT (Month 9) and OLT (Month 13.5), and **(b)** overall mean change in younger (<61 years) versus older (61–68 years) patients, as estimated from MMRM. MMRM model adjusted for baseline domain score, treatment, country, visit, visit-by-treatment interaction, age group, and age-group-by-treatment interaction. EPIC scores range from 0–100; higher scores indicate better values.

**Figure 2:**
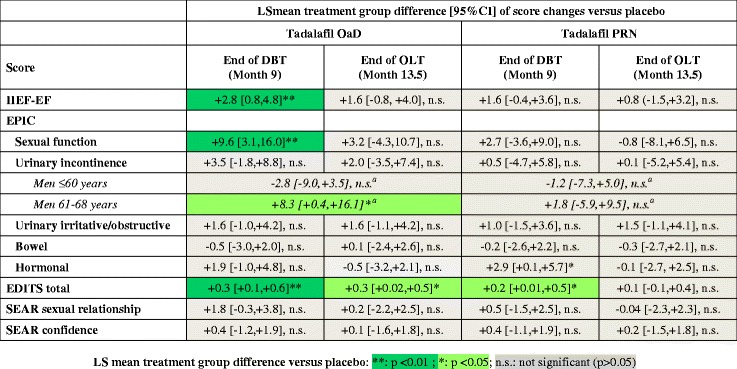
Summary of treatment group differences between tadalafil OaD and tadalafil PRN versus placebo at the end of DBT and OLT. ^a^For men ≤ 60 years and 61–68 years, the overall treatment effect presented includes all visits from baseline to end of OLT. Data are from MMRM models, including baseline value (except for EDITS), treatment, country, visit, visit-by-treatment interaction, and age group (men ≤60 years, men 61–68 years). The MMRM assessing the combined sexual/incontinence score (post-hoc) additionally adjusted for body mass index, smoking status, nerve-sparing score, and type of surgery (open, conventional, robot-assisted, other). Age-group-by-treatment interaction was included in the models only if significant at the 10% level.

EPIC sexual and urinary domain scores improved in all 3 treatment groups during DBT and continued to improve during OLT (Table 2). EPIC sexual domain scores improved significantly with tadalafil OaD versus placebo at the end of DBT (Figure 1; treatment group difference [95% CI]: 9.6 [3.1, 16.0]; p = 0.004), but not with tadalafil PRN versus placebo. The difference between groups was no longer significant at the end of OLT, i.e. after all patients had received tadalafil OaD treatment for 3 months (3.2 [−4.3, 10.7]; p = 0.406). There was no significant difference in EPIC domain scores between the PRN and placebo group at the end of DBT (Figures 1,2).

**Table 2:** **LS mean changes [95%CI] in EPIC domain scores from baseline**

	**Tadalafil OaD (N = 139)**	**Tadalafil PRN (N = 142)**	**Placebo (N = 141)**
**EPIC sexual domain (age-group by treatment interaction: p = 0.083)** ^**a**^			
End of DBT	+27.5 [21.6, 33.4]**	+20.7 [15.3, 26.1]	+18.0 [12.1, 23.8]
End of OLT	+36.6 [30.0, 43.1]	+32.6 [26.6, 38.6]	+33.4 [27.0, 39.8]
*Men ≤60 years*	*+30.1 [23.2, 36.9]*	*+31.2 [24.8, 37.6]*	*+24.9 [18.2, 31.6]*
*Men 61–68 years*	*+34.0 [26.0, 42.0]*	*+22.1 [14.6, 29.5]*	*+26.5 [18.5, 34.4]*
**EPIC urinary incontinence domain (age-group by treatment interaction: p = 0.084)** ^**a**^			
End of DBT	+34.1 [29.3, 38.9]	+31.1 [26.7, 35.5]	+30.6 [25.9, 35.3]
End of OLT	+37.4 [32.6, 42.3]	+35.5 [31.1, 40.0]	+35.4 [30.7, 40.2]
*Men ≤60 years*	*+33.0 [27.7, 38.3]*	*+34.6 [29.6, 39.7]*	*+35.8 [30.5, 41.1]*
*Men 61–68 years*	+38.5 [32.2, 44.8]*	*+32.0 [26.2, 37.9]*	*+30.2 [24.0, 36.5]*
**EPIC urinary irritative/obstructive domain**			
End of DBT	+13.8 [11.5, 16.1]	+13.3 [11.2, 15.4]	+12.3 [10.0, 14.5]
End of OLT	+13.9 [11.5, 16.2]	+13.8 [11.7, 15.9]	+12.3 [10.0, 14.6]
**EPIC bowel domain**			
End of DBT	+5.9 [3.7, 8.2]	+6.3 [4.2, 8.3]	+6.5 [4.3, 8.7]
End of OLT	+6.9 [4.7, 9.1]	+6.5 [4.5, 8.5]	+6.8 [4.6, 8.9]
**EPIC hormonal domain**			
End of DBT	+1.7 [−0.8, 4.3]	+2.7 [0.4, 5.1]*	−0.2 [−2.7, 2.3]
End of OLT	+2.5 [0.1, 4.9]	+2.9 [0.8, 5.1]	+3.0 [0.7, 5.4]

**p < 0.01, *p < 0.05 versus placebo (MMRM).

^a^Significant at the 10% level.

*Abbreviations:* *CI* confidence interval, *DBT* double-blind treatment, *EPIC* Expanded Prostate Cancer Index Composite (EPIC-26), *LS mean* least squares mean, *MMRM* mixed model for repeated measures, *N* number of patients in the ITT population, *OaD* once daily, *OLT* open-label treatment, *PRN* “pro-re-nata”/on-demand.

Data are from MMRM, including baseline domain score, treatment, country, visit, visit-by-treatment interaction, and age group (men ≤60 years, men 61–68 years) (combined sexual/ incontinence score: additionally adjusted for body mass index, smoking status, nerve-sparing score, and type of surgery [open, conventional, robot-assisted, other]). Age-group-by-treatment interaction was included only if significant at the 10% level. For men ≤60 years and 61–68 years (data shown in italics), the overall treatment effect presented includes all visits from baseline to end of OLT.

No significant group differences between tadalafil OaD and placebo were observed for the other EPIC domain scores (Figure 2).

**Figure 3:**
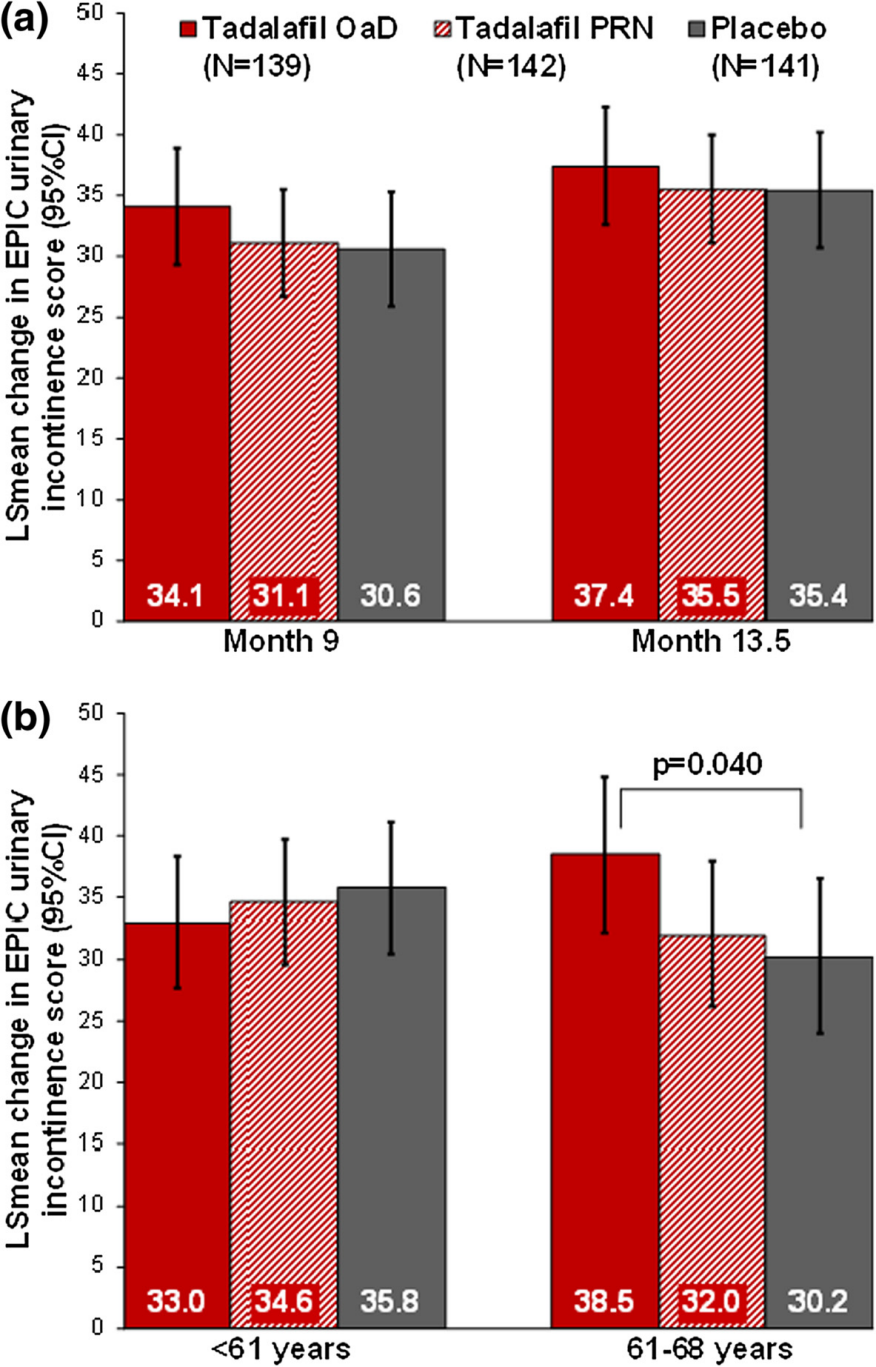
EPIC Urinary Incontinence Score Changes from Baseline: **(a)** at the end of DBT (Month 9) and OLT (Month 13.5), and **(b)** overall mean change in younger (<61 years) and older (61–68 years) patients, as estimated from MMRM. Data from MMRM model, adjusting for baseline domain score, treatment, country, visit, visit-by-treatment interaction, age group, and age-group-by-treatment interaction. EPIC scores range from 0–100; higher scores indicate better values.

A significant interaction (p ≤ 0.1) between age group and treatment was observed for EPIC sexual (p = 0.083) and urinary incontinence (p = 0.084) domain scores. In older patients (61-68 years), EPIC urinary incontinence domain scores improved significantly with tadalafil OaD versus placebo (Figure 3; overall treatment effect across all visits: 8.3 [0.4, 16.1]; p = 0.040). Unadjusted EPIC domain score data (Additional file 1: Table S2) were consistent with these findings.

### EPIC domain scores – patient-partner agreement

Approximately one-third (N = 153) of patients’ partners attended the study visits and completed the EPIC partner questionnaire. Patient-partner agreement could only be assessed for approximately one-third of patients (e.g., partner and patient baseline EPIC sexual domain score available for 140 of 422 patients, 33.2%). Agreement between patients and partners’ ratings was poor (0 to 0.2) to moderate (0.4 to 0.6) for the different time points and domains assessed; no definite pattern of agreement was observed.

### Treatment satisfaction

**Figure 4:**
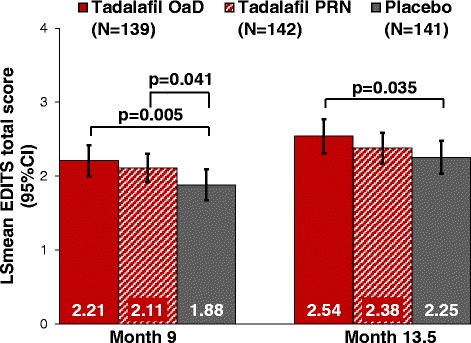
EDITS Total Scores at the End of DBT (Month 9) and OLT (Month 13.5). Data from MMRM model, adjusting for treatment, country, visit, visit-by-treatment interaction, age group, and age-group-by-treatment interaction. EDITS total score ranges from 0–4; higher score indicates better value.

Treatment satisfaction (EDITS total scores) increased significantly in both tadalafil groups when compared with placebo at the end of DBT (OaD versus placebo: 0.33 [0.10,0.56]; p = 0.005, and PRN versus placebo: 0.23 [0.01,0.45]; p = 0.041) (Figure 2,4). At the end of OLT, improvement was only significant for tadalafil OaD versus placebo (p = 0.035). Unadjusted data for EDITS total scores were consistent (Additional file 1: Table S3).

No significant treatment group differences were observed for SEAR (Additional file 1: Tables S4 and S5).

## Discussion

Any major surgery is expected to reduce overall patient QoL during the initial stages of healing and rehabilitation, this is enhanced by the psychosocial impact of a cancer diagnosis. Patients after nsRP face additional challenges, namely impaired EF and urinary continence, which may continue to affect their QoL long after the initial healing phase is complete [17,18]. This can be particularly difficult for younger patients who may be more sexually active than the elderly population. Patients are faced with a long period of time during which sexual and urinary function is not entirely regained, substantially impacting QoL not only for the patients recovering from nsRP but also for their partners [19].

As reported previously, 9 months of DBT with tadalafil OaD, but not tadalafil PRN, significantly increased and accelerated EF recovery when compared with placebo [9,20]; improvements in IIEF-EF and SEP-3 exceeded the minimum clinically relevant difference (MCID) [9]. Treatment with tadalafil OaD treatment was well tolerated; no new safety signals were detected in the prostate cancer patient population [9]. This analysis evaluated if tadalafil treatment affected the patients’ perceived QoL in terms of sexual function and urinary incontinence, as assessed by the respective EPIC domain scores. During DBT, IIEF-EF, EPIC sexual domain score, and EDITS score improved with tadalafil OaD versus placebo but not with tadalafil PRN (Figure 2). This may be due to chronic (daily) tadalafil dosing that would lead to steady-state PDE5-inhibition [21] and may be associated with prolonged (continuous) periods of increased tissue oxygenation during the post-operative regenerative process.

During DBT, patient-rated EPIC sexual domain scores improved in all 3 treatment groups by 27.5% with tadalafil OaD, 20.7% with tadalafil PRN, and 18.0% with placebo. As for IIEF-EF and SEP-3 [9], the improvement was statistically significant versus placebo (p = 0.004) in the tadalafil OaD group only.

The LS mean difference in EPIC sexual domain score changes between tadalafil OaD and placebo was 3.2 points; there is currently no general consensus on how to define the MCID for EPIC domain scores. During the 3 months of additional OLT with tadalafil OaD, EPIC sexual domain scores continued to improve, with overall improvements of more than 30% from baseline to the end of OLT in all 3 groups. The significant treatment group differences from DBT were not maintained as all groups received active tadalafil OaD during OLT.

A significant age group-by-treatment interaction (p = 0.084) indicated that in older patients only (61–68 years of age), EPIC urinary incontinence scores also significantly improved with tadalafil OaD compared with placebo (no significant effect with PRN). The effect was not observed in younger patients (<61 years of age), potentially because they were less affected by urinary incontinence symptoms. There are currently competing hypotheses for the etiology of post-prostatectomy incontinence [22]. Apart from the experience of the surgeon and the surgical technique employed, the most relevant pre-operative predictors of urinary incontinence following robot-assisted nsRP as identified by a meta-analysis included age, body mass index (BMI), comorbidity index, lower urinary tract symptoms, and prostate volume [23]. Urinary incontinence after nsRP is known to be a long-term complication. In the prostate cancer outcome study, 8.4% of all patients were incontinent at ≥18 months after nsRP, even more (14%) had urinary incontinence symptoms after 5 years [12,17]. In a recent study by Nam et al. approximately 5% of patients required incontinence surgery within 15 years post-nsRP [24]. In both studies, the risk for late urinary complications post-nsRP also increased with age [17,24]. In the elderly patients, a fair correlation between EPIC urinary and sexual domain scores (highest at baseline: r = 0.39) was observed in the current study.

Several clinical trials show that PDE5 inhibitors, including tadalafil OaD, can reduce lower urinary tract symptoms in patients with benign prostate hyperplasia [25-27]. A positive effect for PDE5 inhibitors on urinary continence following nsRP was initially observed in a retrospective study by Gandaglia et al. [28]. More recently, Gacci et al. reported significantly improved urinary continence in patients using vardenafil (nightly) compared with placebo in a prospective 12-month study (39 patients) [29].

It is not fully understood how PDE5 inhibitors might act to improve bladder function, although improvements in sphincteric and/or pelvic floor blood supply could be responsible for this effect [28,29]. Moreover, the human bladder expresses high levels of PDE5, inhibition of which can modulate bladder contractility through induction of cyclic guanosine monophosphate [29,30]. The correlation between EPIC urinary incontinence and sexual domain scores decreased over time, potentially due to a differential improvement of sexual and urinary scores.

The perception of the partner during couples’ sexual recovery after nsRP has not been extensively studied, although partners’ needs should be addressed as legitimate aspect of patient care [31]. In this study, only 33.2% of patients’ partners completed the EPIC questionnaire, no clear pattern of patient-partner agreement could be derived. Findings in other studies have indicated that partner QoL can be negatively impacted for at least as long as a patient’s QoL following prostate cancer treatment [19,32].

Although significant improvements in the EPIC sexual and urinary incontinence domains were only observed for tadalafil OaD treatment, treatment satisfaction at the end of DBT, as measured by the EDITS questionnaire, was significantly improved following both tadalafil OaD and PRN administration relative to placebo. However, at the end of OLT, improvement in treatment satisfaction was only significant for tadalafil OaD versus placebo.

There were several potential limitations of the current study. One important consideration is that the patient group selected may not have been ideal for observing an effect on EF or urinary incontinence with chronic tadalafil administration. Patients were on average relatively young (58 years of age), sexually active, and had few comorbidities, thus possibly representing a population of patients who might have shown improvement of EF and urinary continence rates after nsRP without treatment [33]. This could result in dilution of an effect of tadalafil on QoL, and might explain why tadalafil OaD administration significantly improved EPIC urinary incontinence domain scores versus placebo in the elderly population (61 to 68 years) only. Thus, future studies with different patient populations (older, less fit) could help clarify the effect of tadalafil OaD treatment on QoL in post-nsRP patients.

Additional limitations were imposed by the study design. First, the 9-month DBT period may have been too short for optimal assessment of EF recovery, sexual function, urinary incontinence, and associated QoL. After 9 months of DBT, low EF recovery rates of 25.2%, 19.7%, and 14.2% for tadalafil OaD, tadalafil PRN, and placebo, respectively, were observed [9]. In contrast, a retrospective study from Briganti et al. found 3-year EF recovery rates following nsRP of 72% in patients receiving PDE5 inhibitors versus 38% in patients receiving placebo [1], and a recent sildenafil study found recovery rates of around 40% following 12 months of treatment [34]. Second, EPIC scores were not assessed at the end of the drug-free washout period, but only at the end of DBT and OLT. Thus, sexual function and urinary incontinence after treatment cessation could not be assessed. Also, no valid conclusions are possible regarding the patient-partner agreement for EPIC domain scores since only one-third of the partners completed the questionnaire. Finally, the only source for the data on urinary incontinence was the respective EPIC domain score. EPIC data were collected to evaluate patients’ disease-specific QoL; standard instruments for assessment of urinary function, such as the International Prostate Symptom Score might be better suited to evaluate the impact of PDE5 inhibitor treatment on urinary function.

## Conclusion

Chronic dosing of tadalafil started early after nsRP increases and accelerates EF recovery [9,20] and also improves patients’ QoL. The improvement of urinary incontinence facilitated by tadalafil OaD specifically in elderly patients may contribute to this effect on QoL.

## Additional file

Additional file 1:
**Table S1.** List of Ethical Review Boards. **Table S2.** Arithmetic mean EPIC domain score changes (patient). **Table S3.** Arithmetic mean EDITS total scores. **Table S4.** LSmean changes [95% CI] in SEAR domain scores from baseline. **Table S5.** Arithmetic mean SEAR scores. **Figure S1.** Trial design. **Figure S2.** Patient disposition.

## Abbreviations

CI: Confidence interval
DBT: Double-blind treatment
ED: Erectile dysfunction
EDITS: Erectile Dysfunction Inventory of Treatment Satisfaction
EF: Erectile function
EPIC: Expanded Prostate Cancer Index Composite (EPIC-26)
IIEF: International Index of Erectile Function
LS mean: Least squares mean
MMRM: Mixed model for repeated measures
N: Number of patients in the ITT population
nsRP: Nerve-sparing radical prostatectomy
OaD: Once daily
OLT: Open-label treatment
PRN: “Pro-re-nata”/on-demand
QoL: Quality of life
SEP: Sexual Encounter Profile
SEAR: Self-Esteem and Relationship
