# Supplementary material for: Effects of tadalafil treatment after bilateral nerve-sparing radical prostatectomy: quality of life, psychosocial outcomes, and treatment satisfaction results from a randomized, placebo-controlled phase IV study
Source: BMC Urol. 2015 Apr 12;15:31. doi: 10.1186/s12894-015-0022-9 (PMC4419565; doi:10.1186/s12894-015-0022-9)
Supplement: Additional file 1: — Table S1. List of Ethical Review Boards. Table S2. Arithmetic mean EPIC domain score changes (patient). Table S3. Arithmetic mean EDITS total scores. Table S4. LSmean changes [95% CI] in SEAR domain scores from baseline. Table S5. Arithmetic mean SEAR scores. Figure S1. Trial design. Figure S2. Patient disposition. [file 12894_2015_22_MOESM1_ESM.docx]

# Supplemental Material

**Supplemental Table S1: List of Ethical Review Boards**

| **Country** | **Ethical Review Board** | **City** | **Zip/ Post** | **Address** |
| --- | --- | --- | --- | --- |
| BELGIUM | Comité d'éthique,  Centre Hospitalier Universitaire Sart Tilman | Liege | 4000 | Batiment B-35 Sart Tilman par Liege 1 |
| BELGIUM | Commissie voor Medische Ethiek, Univ Ziekenhuis Gasthuisberg Leuven | Leuven | 3000 | Herestraat 49 |
| BELGIUM | Ethisch Comité, AZ Groeninge, / West-Vlaanderen | Kortrijk | 8500 | Burg. J. Vercruysselaan 5 |
| FRANCE | CPP SUD Mediterranee III | Nimes Cedex 2 | 30908 | Av Kennedy, CS 83021 |
| CANADA | Office of Research Ethics, University of Western Ontario | London | N6A 5C1 | Support Building,  Room 4180 |
| CANADA | Conjoint Medical Ethics Committee, University of Calgary | Calgary | T2N4N1 | 3330 Hospital Drive NW |
| CANADA | Institutional Review Board Services, Aurora, Ontario | Aurora | L4G 0A5 | 372 Hollandview Trail |
| GERMANY | 09/0414-ZSEK13, Landesamt für Gesundheit und Soziales (LAGeSo) | Berlin | 10707 | Fehrbelliner Platz 1 |
| ITALY | Comitato Etico, Instituto Scientifico Universitario San Raffaele | Milano | 20132 | Via Olgettina, 60 |
| ITALY | Comitato Etico, Ospedale San Martino Universita' Genova | Genova | 16132 | Largo Rosanna Benzi, 10 |
| ITALY | Comitato Etico, CEAS Umbria | Ellera di Corciano | 06074 | Via del la Rivoluzione,16 |
| ITALY | Comitato Etico, Ospedale Miull | AcquavivadelleFonti | 70021 | Prov. Acquaviva - Santeramo Km. 4,100 |
| ITALY | Comitato Etico Indipendente, Ospedale Malpighi | Bologna | 40138 | Via Albertoni, 15 |
| ITALY | Comitato Etico, Ospedali Riuniti di Bergamo | Bergamo | 24128 | Largo Barozzi, 1 |
| NETHERLANDS | CMO regio Arnhem-Nijmegen, H6D-EW-LVIK, 2009-011850-17 CMO 2009/206 | Nijmegen | 6500 HB | UMC St Radboud Huispostnummer 578, Postbus 9101 |
| POLAND | SW IZBA LEKARSKA KOMISJA BIOETYKI, | KIELCE | 25-389 | UL. WOJSKA POLSKIEGO 52 |
| SPAIN | Comité Ético de investigación, Hosptial Puerta De Hierro | Majadahonda | 28222 | Manuel de Falla, 1 |
| SPAIN | Comité Ético de Investigación, Hosptial Puerta De Hierro | Majadahonda | 28222 | Manuel de Falla, 1 |
| SPAIN | CEIC Autonómico de Ensayos Clínicos de Andalucía, Comite Etico de Ensayos Clinicos CAEC | Sevilla | 41080 | Avda. de la Innovacion s/n, Ed. Arenal 1 |
| SPAIN | CEIC area 5, Hospital La Paz : Dª Paz Lavilla / Dª Emma Fernández de Uzquiano | Madrid | 28046 | Paseo De La Castellana, 261 |
| SPAIN | CEIC area 11, Hospital Doce de Octubre Dª María Ugalde Díez | Madrid | 28041 | Avda de Cordoba |
| SPAIN | Comité Ético de Investigación Clínica, Hospital General Universitario Gregorio Marañon | Madrid | 28007 | Doctor Esquerdo, 46 |
| SPAIN | Comité Etico de Investigación Clínica, Universitario de Valencia Hospital | Valencia | 46010 | Avda Blasco Ibanez, 17 |
| SPAIN | Comité Ético de Invesetigación Clínica, Complejo Hospitalario Carlos Haya | Malaga | 29010 | Avda. Carlos Haya, s/n |
| Switzerland | Kantonale Ethik Kommission Zürich | Zurich | 8090 | Stampfenbachstrasse 121 |
| Switzerland | Ethikkommission des Kantons Luzern | Luzern | 3439 | Meyerstrasse 20, Postfach 3439 |
| UNITED KINGDOM | ERB South Central - Oxford C | Jarrow | NE32 3DT | TEDCO Business Centre Room 002 Rolling Mill Road |
| UNITED KINGDOM | ERB - Oxford B Research Ethics Committee | Bicester | OX26 4JT | Astral House, Chaucer Business Park, Granville Way |

Supplemental Table S2: Arithmetic mean EPIC domain score changes (patient)

|  | Tadalafil OaD  (N=139) | | Tadalafil PRN (N=142) | | Placebo (N=141) | |
| --- | --- | --- | --- | --- | --- | --- |
|  | n | Mean (SD) | n | Mean (SD) | n | Mean (SD) |
| EPIC sexual domain score | | | | | | |
| Baseline | 133 | 19.8 (19.56) | 140 | 21.9 (20.16) | 137 | 20.1 (21.87) |
| Change at end of DBT | 102 | +25.6 (24.67) | 113 | +20.7 (23.85) | 108 | +16.9 (21.92) |
| Change at end of OLT | 94 | +34.0 (25.61) | 108 | +32.8 (26.99) | 101 | +31.2 (29.31) |
| EPIC urinary incontinence domain score | | | | | | |
| Baseline | 133 | 46.7 (30.71) | 139 | 47.9 (28.89) | 137 | 49.5 (28.05) |
| Change at end of DBT | 101 | +32.3 (26.44) | 111 | +32.5 (25.18) | 104 | +27.9 (23.84) |
| Change at end of OLT | 93 | +34.4 (29.19) | 106 | +36.4 (27.14) | 99 | +32.7 (25.44) |
| EPIC urinary irritative/obstructive domain score | | | | | | |
| Baseline | 131 | 78.1 (18.92) | 137 | 81.5 (15.08) | 134 | 81.3 (16.42) |
| Change at end of DBT | 97 | +15.5 (19.03) | 107 | +12.0 (15.94) | 101 | +10.6 (15.02) |
| Change at end of OLT | 91 | +15.8 (19.75) | 104 | +12.5 (15.89) | 95 | +10.7 (14.92) |
| EPIC bowel domain score | | | | | | |
| Baseline | 129 | 88.3 (15.32) | 134 | 91.2 (10.31) | 136 | 89.9 (13.69) |
| Change at end of DBT | 95 | +6.7 (15.83) | 105 | +4.4 (11.87) | 106 | +6.3 (13.20) |
| Change at end of OLT | 89 | +8.1 (16.67) | 101 | +4.8 (12.81) | 96 | +6.4 (13.39) |
| EPIC hormonal domain score | | | | | | |
| Baseline | 130 | 90.0 (12.71) | 137 | 92.0 (10.51) | 136 | 91.4 (10.89) |
| Change at end of DBT | 98 | +1.3 (16.08) | 107 | +2.3 (9.31) | 104 | -0.6 (12.13) |
| Change at end of OLT | 91 | +1.8 (14.75) | 105 | +2.3 (11.38) | 97 | +2.6 (9.82) |

Abbreviations: DBT, double-blind treatment; EPIC, Expanded Prostate Cancer Index Composite (EPIC-26); N, number of patients in the ITT population; n, number of patients with data; OaD, once daily; OLT, open-label treatment; PRN, “pro-re-nata”/on-demand; SD, standard deviation

EPIC domain scores were computed based on individual items standardized to a 0‑100 scale.

Supplemental Table S3: Arithmetic mean EDITS total scores

|  | Tadalafil OaD  (N=139) | | Tadalafil PRN (N=142) | | Placebo (N=141) | |
| --- | --- | --- | --- | --- | --- | --- |
|  | n | Mean (SD) | n | Mean (SD) | n | Mean (SD) |
| End of DBT | 95 | 2.3  (0.86) | 116 | 2.1  (0.85) | 104 | 1.9  (0.80) |
| End of OLT | 92 | 2.5  (0.93) | 109 | 2.4  (0.92) | 101 | 2.3  (1.00) |

Abbreviations: DBT, double-blind treatment; EDITS, Erectile Dysfunction Inventory of Treatment Satisfaction; N, number of patients in the ITT population; n, number of patients with data; OaD, once daily; OLT, open-label treatment;
PRN, “pro-re-nata”/on-demand; SD, standard deviation
EDITS total score, range from 0-4; higher score indicates better value.

Supplemental Table S4: LSmean changes [95%CI] in SEAR domain scores from baseline

|  | LSmean changes [95%CI] | | | p-value | | |
| --- | --- | --- | --- | --- | --- | --- |
|  | Tadalafil OaD (N=139) | Tadalafil PRN (N=142) | Placebo (N=141) | OaD vs. placebo | PRN vs. placebo | Overall |
| Sexual relationship domain (score range 8-40) | | | | | | |
| End of DBT | +4.78 [2.87, 6.68] | +3.50 [1.61, 5.38] | +3.02 [1.09, 4.95] | 0.086 | 0.637 | 0.269 |
| End of OLT | +6.90 [4.79, 9.00] | +6.68 [4.62, 8.74] | +6.72 [4.62, 8.83] | 0.885 | 0.972 |  |
| Confidence domain (score range 6-30) | | | | | | |
| End of DBT | +1.28 [-0.27, 2.84] | +1.29 [-0.25, 2.82] | +0.89 [-0.69, 2.47] | 0.616 | 0.605 | 0.733 |
| End of OLT | +2.56 [0.92, 4.20] | +2.62 [1.02, 4.21] | +2.44 [0.80, 4.09] | 0.895 | 0.835 |  |
| Self-esteem subdomain (score range 4-20) | | | | | | |
| End of DBT | +0.78 [-0.31, 1.87] | +0.79 [-0.29, 1.87] | +0.51 [-0.61, 1.62] | 0.621 | 0.598 | 0.693 |
| End of OLT | +1.67 [0.54, 2.81] | +1.72 [0.61, 2.83] | +1.62 [0.47, 2.77] | 0.923 | 0.857 |  |
| Overall relationship subdomain (score range 2-10) | | | | | | |
| End of DBT | +0.56 [-0.11, 1.23] | +0.58 [-0.08, 1.24] | +0.47 [-0.20, 1.15] | 0.782 | 0.741 | 0.896 |
| End of OLT | +0.96 [0.28, 1.64] | +1.01 [0.35, 1.67] | +0.90 [0.22, 1.57] | 0.858 | 0.720 |  |

Abbreviations: CI, confidence interval; DBT, double-blind treatment; LSmean, least squares mean; N, number of patients in the ITT population; OaD, once daily; OLT, open-label treatment; PRN, “pro-re-nata”/on-demand; SEAR, Self-Esteem and Relationship Questionnaire
p-values were obtained from repeated measures analysis of the change from baseline in the SEAR score, with baseline domain score, treatment, country, visit, visit-by-treatment interaction, age group, age group-by-treatment interaction.

Supplemental Table S5: Arithmetic mean SEAR scores

|  | Tadalafil OaD  (N=139) | | Tadalafil PRN (N=142) | | Placebo (N=141) | |
| --- | --- | --- | --- | --- | --- | --- |
|  | n | Mean (SD) | n | Mean (SD) | n | Mean (SD) |
| Sexual relationship domain (score range 8-40) | | | | | | |
| Baseline | 134 | 17.7 (7.73) | 136 | 18.0 (7.87) | 136 | 17.5 (7.93) |
| Change at end of DBT | 103 | +5.7 (8.59) | 111 | +4.6 (7.78) | 106 | +3.8 (8.42) |
| Change at end of OLT | 91 | +7.6 (9.44) | 104 | +7.5 (9.37) | 100 | +7.3 (9.71) |
| Confidence domain (score range 6-30) | | | | | | |
| Baseline | 135 | 20.3 (6.71) | 139 | 20.0 (6.27) | 136 | 20.0 (7.22) |
| Change at end of DBT | 104 | +0.6 (7.03) | 114 | +0.8 (6.45) | 106 | +0.5 (6.18) |
| Change at end of OLT | 93 | +1.9 (7.91) | 106 | +2.1 (6.95) | 100 | +2.1 (7.42) |
| Self-esteem subdomain (score range 4-20) | | | | | | |
| Baseline | 137 | 13.6 (4.67) | 140 | 13.3 (4.55) | 136 | 13.3 (4.92) |
| Change at end of DBT | 106 | +0.3 (4.99) | 115 | +0.5 (4.48) | 106 | +0.3 (4.24) |
| Change at end of OLT | 96 | +1.2 (5.39) | 107 | +1.4 (4.86) | 100 | +1.5 (5.07) |
| Overall relationship subdomain (score range 2-10) | | | | | | |
| Baseline | 135 | 6.7 (3.06) | 139 | 6.7 (2.84) | 138 | 6.7 (3.11) |
| Change at end of DBT | 104 | +0.2 (3.29) | 114 | +0.3 (3.08) | 108 | +0.2 (3.01) |
| Change at end of OLT | 93 | +0.7 (3.56) | 107 | +0.7 (3.11) | 102 | +0.6 (3.23) |

Abbreviations: DBT, double-blind treatment; N, number of patients in the ITT population; n, number of patients with data; OaD, once daily; OLT, open-label treatment; PRN, “pro‑re‑nata”/on-demand; SD, standard deviation; SEAR, Self-Esteem and Relationship Questionnaire

Supplemental Figure S1: Trial design

Abbreviations: nsRP, bilateral nerve-sparing radical prostatectomy; d, day; mo, month;
OaD, once daily; PRN, “pro-re-nata”/on-demand; V, visit; wk, week

Supplemental Figure S2: Patient disposition

One subject assigned to tadalafil PRN group did not receive any study drug and was therefore not included in the analysis of the PRN group.

Abbreviations: OaD = once daily; PRN = “pro-re-nata”/on-demand; N = number of patients
